# Self-management of male urinary symptoms: qualitative findings from a primary care trial

**DOI:** 10.3399/BJGP.2025.0046

**Published:** 2026-02-10

**Authors:** Jessica R Wheeler, Jo Worthington, Marcus J Drake, Jessica Frost, Mandy Fader, Lucy McGeagh, Hashim Hashim, Margaret Macaulay, Jonathan Rees, Luke A Robles, Gordon Taylor, Matthew J Ridd, Stephanie J MacNeill, Sian Noble, J Athene Lane, Nikki Cotterill

**Affiliations:** 1 Bristol Medical School, University of Bristol, Bristol, UK; 2 NIHR Applied Research Collaborations West, Bristol, UK; 3 Faculty of Medicine, Imperial College London, London, UK; 4 School of Health Sciences, University of Southampton, Southampton, UK; 5 Oxford Institute Nursing, Midwifery and Allied Health Research, Oxford Brookes University, Oxford, UK; 6 Bristol Urological Institute, North Bristol NHS Trust, Bristol, UK; 7 Tyntesfield Medical Group, Brockway Medical Centre, Bristol, UK; 8 Nuffield Department of Primary Care Health Sciences, University of Oxford, Oxford, UK; 9 Public and patient involvement representative, Oxford, UK; 10 School of Health and Social Wellbeing, University of the West of England, Bristol, UK

**Keywords:** general practice, primary care, qualitative interviews, randomised controlled trial, self-management, lower urinary tract symptoms

## Abstract

**Background:**

Informed self-management is the first-line treatment for male lower urinary tract symptoms (LUTS), although the extent of delivery in primary care is unclear. The TReating Urinary symptoms in Men in Primary Health care (TRIUMPH) cluster randomised controlled trial (reference: ISRCTN11669964) compared a structured self-management intervention with usual care for men with LUTS. We report on embedded qualitative interviews.

**Aim:**

To investigate men’s experiences of LUTS, engagement with primary care, and responses to a self-management intervention, along with the perspectives of primary care clinicians in order to inform the delivery of self-management guidance in primary care.

**Design & setting:**

Qualitative interview study embedded in the TRIUMPH trial, which was conducted across 30 general practice sites in the South West of England.

**Method:**

Semi-structured interviews were conducted with 58 men with LUTS (selected purposively from the TRIUMPH trial population) and 14 treating clinicians (recruited from the TRIUMPH trial primary care sites), then analysed using thematic analysis.

**Results:**

Men with LUTS were characterised as ‘languishing’, poorly informed, discounting symptoms as ‘just old men’s problems’, and experiencing prostate-specific antigen (PSA) testing cycles that did not resolve their LUTS. GPs described a focus on LUTS self-management being restricted by clinical pressures and attending to prostate cancer concerns. The TRIUMPH self-help intervention booklet was strongly valued by men: many reported that it gave them a greater understanding of their symptoms and self-management options, reduced anxiety, and that using it improved their LUTS and quality of life. A few men, however, found the intervention unrewarding.

**Conclusion:**

Explanations and tailored self-management support were liked and found useful by many men with LUTS. Recommendations for clinical practice include: avoiding the expression ‘old men’s problems’, ensuring LUTS follow-up after PSA testing, focusing on symptoms and self-management approaches, and distributing the TRIUMPH booklet widely.

## How this fits in

Male lower urinary tract symptoms (LUTS) cause a marked negative impact on daily life, but men do not readily seek help. Self-management for LUTS in men is well received in primary care and increases men’s agency, however opportunities for LUTS education and improvement are easily missed in the prostate-specific antigen testing cycle for prostate cancer. Dissemination and delivery of the TReating Urinary symptoms in Men in Primary Health care (TRIUMPH) booklet was strongly advocated by the men involved in this study.

## Introduction

Lower urinary tract symptoms (LUTS) are common in older men (typically over the age of 50 years) and as symptoms worsen (from mild, to moderate or severe, depending on the range, frequency, intensity and/or distress that is reported in relation to a man’s LUTS), they can strongly impact quality of life.^1–9^ LUTS include those related to storage (increased frequency, urgency, nocturia), voiding (slow stream, hesitancy), and post-voiding issues (dribbling, incomplete emptying). Causes include prostatic obstruction, overactive bladder syndrome, detrusor dysfunction, and lifestyle factors.^10–13^ As symptoms worsen, the negative effects on mental health and wellbeing intensify; these range from sleep disturbance and restricted activities to anxiety and low self-esteem.^2,14,15^ Prevalence estimates for male LUTS are considerably larger than the number of men who are clinically treated .^16,17^

Men typically first present with LUTS in primary care. Referrals to specialist urology services are common, resulting in calls to expand and improve symptom management in primary care.^18^ Established treatments range from lifestyle, pharmacological, and surgical interventions,^10^ with the evidence base weighted towards medication and surgery,^19^ although side-effects, non-adherence, surgical risks, and enduring symptoms are common.^20–25^ Although less studied, informed self-management — including pelvic floor exercises, dietary/fluid guidance, urethral bulb compression and release, and urination advice — offer targeted symptom-focused approaches.^10,26,27^ These strategies minimise side-effects^26,28–30^ and are recommended as first-line treatments for men with uncomplicated LUTS.^10^

Prior qualitative work in primary care suggests that men with LUTS are often neither well informed about their condition, nor aware of appropriate self-management techniques.^18,31^ As such, research on the nature, quality, and delivery of self-management guidance in primary care is warranted.^18^ The national TReating Urinary symptoms in Men in Primary Health care (TRIUMPH) cluster randomised controlled trial (RCT) (reference: ISRCTN11669964) was conducted to determine whether a standardised approach to LUTS self-management guidance in primary care improved outcomes in comparison with usual care; it utilised a booklet that was co-produced by patients and clinicians, and delivered by healthcare professionals (HCPs).^32–34^ The booklet is available at: bristol.ac.uk/triumph-study/news. The TRIUMPH RCT included a qualitative investigation of experiences of men with LUTS, their clinicians, and the TRIUMPH intervention, to inform delivery of self-management guidance in primary care.^35^

## Method

The qualitative study investigated:

- men in the TRIUMPH trial’s experiences of LUTS care to date and of the intervention or usual care; and
- GPs’ experiences of LUTS consultations and self-management in primary care, as well as HCPs’ experiences of TRIUMPH intervention delivery.

TRIUMPH RCT participants who consented to being approached for interview were purposively selected for the qualitative study on the basis of anonymously recorded demographic and symptom data that was collected from all participating men at the outset of the TRIUMPH RCT. Selected men were identified to the qualitative researcher (by name and contact number) and were contacted by telephone. Those who were contactable within the study timeframe, and were willing and available, were sent additional information about the qualitative study and, with their further informed consent, an interview was arranged. Purposive sampling was undertaken to:

- include men from diverse GP sites and demographics with a wide range of LUTS experiences (including the full range of LUTS, including voiding issues, retention issues, post-voiding dribble, nocturia, bladder sensitivity, and mixed presentation LUTS, at all levels of severity, including men with ‘mild’, ‘moderate’, or ‘severe’ LUTS); and
- allow comparison across intervention and usual-care experiences.

The stages and a timeline of the qualitative interview study, as an embedded component of the TRIUMPH RCT, are outlined in Supplementary Figure 1.^34^ In-depth, semi-structured interviews were conducted in person or by telephone, and audio-recorded and transcribed verbatim. A subset of men with LUTS were interviewed at baseline and follow-up to discuss responses to the intervention over time. Clinicians were interviewed once (GPs to discuss their experiences and treatment of male LUTS; HCPs to discuss their experiences of delivering the intervention). Transcription and coding frames were developed^36^ concurrently using NVivo (version 12) software to inform ongoing interviews.

Themes were reviewed and refined by two experienced qualitative researchers, and presented to the wider project group in line with a thematic analysis approach,^37^ from a critical realist ontological stance.^38^ Reflexivity was an active part of the analytical research process, which played close attention to the role of researcher biases in the shaping of interviews and in their interpretation. This was discussed, with close reference to the qualitative data (interview transcripts), both during research team meetings and with the research team patient advisory group (a group of male urology patients with extensive experience of male LUTS and urology healthcare interventions who were integral to the design of both the TRIUMPH RCT and the structured self-help guidance offered to men in the TRIUMPH RCT, and who provided advice and support as part of research team meetings and dedicated patient advisory group meetings throughout the TRIUMPH RCT). Interviews continued until key codes and themes addressing the study’s objectives were richly constructed, accounted for discordant perspectives, and were not meaningfully strengthened or challenged through additional interview content.

## Results

### Participant characteristics

Supplementary Table S1 details the characteristics of the men involved in the TRIUMPH qualitative study; the mean age of men in the intervention group was 66 years, whereas that of men in the usual care group was 59 years. Men in both groups were predominantly White British and married, with similar baseline symptom scores. Supplementary Table S2 details the characteristics of clinicians interviewed as part of the TRIUMPH RCT.

### Patient perspectives: identified themes

**Figure 1.**
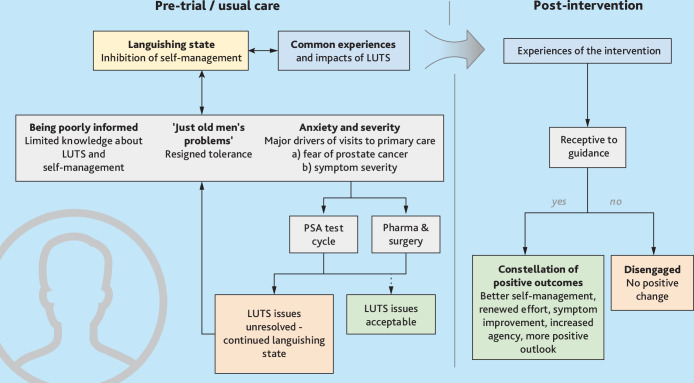
Men with LUTS intervention journey from languishing to self-management: identified themes.

The thematic interpretation of interviews with the participants is set out in Figure 1; quotations illustrating each theme/subtheme are outlined in Table 1.

**Table 1.** Identified themes and corresponding quotations from interviews with men with LUTS

Theme	Quotation
***Pre intervention/usual care***	
Common experiences and impacts of male LUTS	*‘It was bothering me a lot because you are going into work and you are absolutely shattered . . . Tiredness and irritable and being miserable all the time.’ *Intervention group, age 69 years *‘ . . a total nightmare with the wet patches, bursting to go, and having to run to the loo.‘* Intervention group, age 76 years *‘It’s incredibly painful in the morning… What I do get is frustration that I haven’t done it, I haven’t emptied, because I want to get on . . . I want to get on and do other stuff and I keep having to come back and . . . so it’s the time really.’* Control group, age 58 years
The ‘languishing state**’**	*’I just get worn down with it all. I lose all kind of sort of vim and vigour and I can’t do . . . well it stops me from doing the things that I love to do, like going out walking . . . or whatever. You know, that’s a downer as well because I feel I’m sort of hemmed in. I just get fed up. Not quite suicidal but you can get a bit fed up with things.’* Control group, age 62 years *‘I would probably fight shy of bothering the GP with these symptoms, because I would, and probably mistakenly, think it’s not worth bothering them with. Although it’s tiresome to deal with, it’s never seemed to be a medical something you’d go to the doctor about.’* Intervention group, age 69 years *‘My gut feeling is it’s just going to stay like it or get slightly worse and I have to put up with it, really . . . ’* Usual care group, age 66 years *‘I suppose I never articulated this at the time but feeling concerned about a loss of control over my life . . . ‘Loss of control over my life’ sounds rather pretentious but I can’t think of a better way of phrasing it.’* Intervention group, age 78 years
Being poorly informed	’*..maybe I just think I hope it will go away some time. I wouldn’t know whether medically anything can be done about that so I’ve just accepted it as it is.’* Usual care group, age 81 years Interviewer: *‘Did the doctor offer you any advice, or support . . . or anything in relation to the urgency? Any guidance?*’ Interviewee: *’Not that I can remember . . . I was just — possibly for want of a better word — just left to get on with it, I suppose.*’ Usual care group, aged 61 years
‘Just old men’s problems’	*‘* . . . *actually I put it down to getting old.’* Intervention group, aged 68 years *‘Most . . . I’ve been told* [by the GP]*, ”Oh that’s your age. Learn to live with it“. But that’s as far as it went really .’* Intervention group, aged 76 years *‘My recollection of the exercise* [GP consultation] *is ‘That’s what happens with old men’* . . . ’ Intervention group, age 75 years
Anxiety and severity	*‘I sometimes worry that it might be something like sort of cancer, which thankfully it’s proved not to be. That’s when it preys on my mind’.* Usual care group, age 62 years *’Well, it was having to check yourself all the time, I’ve got to go to the loo before I do this. I couldn’t go to a meeting or go out because I’d have to check where the loos were. The whole cycle was making me more and more anxious. It would get to the point where I would say, ‘I’m not doing that. I can’t be bothered doing that. It’s too much stress . . . ’ Usual care group, age 58 years*
The PSA test loop	*’ . . . when you’ve had a PSA test, I know the PSA test is not 100%, but when you’ve had it and it shows a low reading and a prostate examination says it’s okay, there’s a feeling of relief, oh that’s alright I’ll move on. And the symptoms themselves tend to be put into the background . . . ’* Intervention group, aged 75 years
More-severe symptoms and pharmacological pathways	*’ . . . they prescribed me with a daily pill to take . . . I wouldn't say it’s greatly changed. Whether it could do with a further check-up with the GP . . . it’s obviously helping to a certain extent . . . I'm not quite sure?’* Intervention group, aged 76 (prescribed tamsulosin and finasteride by GP)
***Post intervention***	
Constellation of positive outcomes	
Responses to the intervention (overall)	*’I think overall, it’s tremendously reassuring to have this sort of booklet, and to see that all these things are very well recognised and demonstrated and that there are techniques for helping with them.’* Intervention group, aged 69 years
Increased knowledge and awareness	*’I didn’t realise until I got this booklet that men had a pelvic floor, to be quite honest! . . . By just squeezing that muscle, that has also helped . . . me stop having to run to the loo.’* Intervention group, aged 58 years *’Well, I started the exercises that the book gave me . . . hell of a difference . . . I wasn’t dribbling . . . I was only waking up twice a night . . . there was definitely a relief, it helped.*‘ Intervention group, aged 78 years
Symptom improvement	*‘I felt quite embarrassed about it, but now that’s not happening; it’s obviously made me feel quite a bit more cheerful . . . You’re getting old and you think it’s one of the signs of getting old . . . if you can do something about it, it’s quite good for your mental health aspect as well.’* Intervention group, aged 71 years
Wider educational benefits	*’ . . . I have to say that that was probably one of the best things about the book . . . I know a lot of people of my age and a good few of them we’ve all, believe it or not, we’ve actually discussed this, which is unusual for blokes to do that.’* Intervention group, aged 66 years
The role of HCPs	*’I just really appreciate the fact that being given the booklet and being explained and chatted through it with the nurse and everything, it then gave me the motivation to actually start trying things properly and to try it all together and the fact that then it’s made a big improvement.‘* Intervention group, aged 52 years
Empowerment	*‘ . . . I’m less concerned ‘cause I’m feeling I’ve got better control . . . I mean you know, it’s giving yourself control of your own symptoms.’* Intervention group, aged 53 years
Disengaged — little or no positive change	*’There was perhaps a slight relevance to me but not a huge amount . . . It’s not practicable most of the time.*‘ Intervention group, aged 74 years *‘ . . . All the stuff that’s been sent to me, I’ve read it through once. But nothing really was earth-shattering.’* Intervention group, aged 73 years

HCP = healthcare professional. LUTS = lower urinary tract symptoms. PSA = prostrate-specific antigen.

#### Common experiences and effects/impact of male LUTS

Men described LUTS that affected their everyday lives in considerable and familiar ways, with greater disruption experienced by those with more severe symptoms. Impacts included curtailment of everyday activities and diminished mental health associated with anxiety and depression symptoms, reduced wellbeing, and reduced quality of life. Men spoke of embarrassment and shame. For some, the sense of LUTS as an age-related issue was conveyed as something natural that was to be accepted stoically; for others, the association was more stigmatising — especially if symptoms were embarrassing and impossible to hide — and perceived as symbolising an inevitable decline and loss of agency.

The level of disruption men described ranged from frustration and irritation (for example, chronic sleep disturbance, needing to know the location of public toilets) to distress, concern, embarrassment, shame, and anxiety related to leakage, experiences of discomfort, pain, and fear of acute retention. They also reported a pernicious worry about the possibility of underlying cancer.

#### The languishing state

There was an overriding impression, in pre-intervention accounts and throughout the trial for usual care men, of ‘languishing’ — a dispirited, inhibitory state of inaction despite disruptive symptoms, with little hope for future improvement. This was true for men with moderate to severe symptoms, or few and mild symptoms

The languishing state was underpinned by three sub-themes:

- being poorly informed;
- ‘just old men’s problems’; and
- anxiety and severity as the major drivers of primary care visits.

Typically, men had limited awareness of established self-management techniques or could not recall discussing these with their GP, and tended to neglect their LUTS. Men rarely sought in-person clinical support, often doing so only after lengthy delays and increasingly severe symptoms. They sometimes understood their symptoms to be related to enlargement of the prostate as part of ageing; a smaller number described overactive bladder syndrome, incomplete emptying of the bladder, or retention. Knowledge of associated self-management techniques was limited.

Referring to LUTS as ‘old men’s problems’ (see Table 1) was recurrent, and seen to play a dismissive role: it served to encourage men to tolerate LUTS rather than to view them as worthy of action. The notion of ‘old men’s problems’ appeared to reaffirm self-limiting and, at times, highly stigmatised beliefs about age-associated conditions being unworthy of clinical attention and laden with a sense of inevitable decline.

Often when men eventually sought clinical support, it was because their symptoms were increasingly an issue of anxiety or severity, and this had consequences for the nature of the consultation (steering the primary care consultation towards PSA testing or conversely pharmacological treatment, rather than self-help guidance).

Men who described mounting anxiety related to fear of prostate cancer, described attending primary care seeking a prostate-specific antigen (PSA) test. This was associated with a circular pathway, which we have called the PSA test loop. Despite enduring and/or moderate to severe LUTS, men prioritised their concerns about prostate cancer. When results indicated unconcerning PSA levels (often received by telephone), men’s anxieties were alleviated and they were either not offered, and/or did not seek, a follow-up consultation to discuss ongoing LUTS symptoms. This cycle, sometimes repeated several times over the course of years, left the symptoms themselves unattended and unresolved.

For men attending primary care as result of symptom severity, this was often after sustained neglect of increasingly distressing LUTS. In this case, the acute pressure to resolve increasingly severe symptoms led primary care consultations towards pharmacological interventions and/or referral to urology, rather than self-management. Despite varying outcome success and taking medication for lengthy periods (sometimes years), men reported rarely returning to their GP to review their symptoms and medication.

##### Pre-intervention/usual care ‘languishing’: summary

Consultations often focused on PSA testing or medication that did not result in resolution or significant improvements in LUTS, and/or were associated with a continued languishing state (and repeated cycles of PSA testing). A few men reported prior primary care consultations (prior to their participation in the TRIUMPH RCT) that had resulted in some degree of symptom resolution. These men reported being relatively unconcerned about their LUTS at the time of interview. Commonly, men with enduring symptoms — both those who had sought PSA testing and those who had been offered medications — had reached a point of stasis or inaction in relation to unresolved LUTS. While in a ‘languishing state’, men had neither recently revisited, nor intended to revisit, primary care to discuss their ongoing LUTS or to review medications, despite distressing symptoms, even when these were severe.

In summary, the over-arching pre-intervention/usual care theme was that of a ‘languishing state’. For long periods, prior to engagement in the TRIUMPH RCT and enduringly for those in the usual care group, men described a normalised state of dispondency, resigned acceptance, neglect and inaction. This was explained by a series of sub-themes, that described how men were held in, or repeatedly returned men to, a ‘languishing state’. Men’s neglect of their distressing symptoms was bolstered by ‘being poorly informed’ about LUTS and about the possibilities of improvement through self-help, and by their tendencies to dismiss male LUTS as ‘just old men’s problems’ rather than seeking help. The concerns that drove men to take action and to seek primary care support — ‘anxiety’ about the possibility of prostate cancer and/or ‘symptom severity’ — were associated with PSA test cycles that left symptoms unattended, or pharmacological interventions that left symptoms unresolved, returning men recurringly to a ‘languishing state’, without having received targeted self-help guidance .

### Clinician perspectives and integration with patients’ perspectives

**Figure 2.**
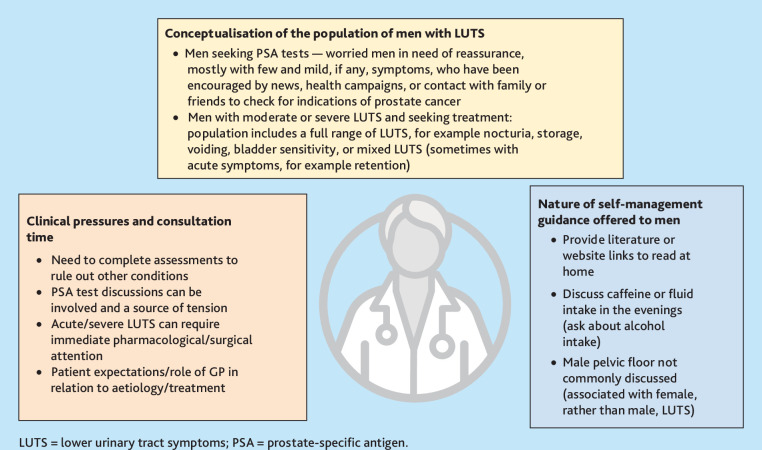
Key themes identified in GP interviews.

GP interviews focused on routine primary care experiences of male LUTS, treatment pathways, and clinical pressures in providing self-management guidance. Figure 2 summarises the dominant themes and arising issues; Table 2 features related quotations from GP interviews.

**Table 2.** GPs’ quotations linked to key themes

Theme	Quotation
***Conceptualisation of the population of men with LUTS***	
Those seeking PSA tests	*‘ . . . I think very few have no symptoms whatsoever but most of the people who are coming asking for PSA* [tests] *I guess perhaps aren't that bothered by their symptoms . . . If you ask about symptoms they say, “Oh no, I’m fine really, I’m normal for my age . . . somebody told me I should have a test . . . ”.’* GP1
Men with moderate-to-severe LUTS	*‘I've never really thought of conservative measures for prostate enlargement* [as opposed to irritable bowel syndrome] *. . . I go straight in with medicines there, possibly too early . . . if they’re not working I would think, “Hang on, this isn’t going the way that I had hoped it would” and I would refer* [to urology]*.’* GP2
***Clinical pressures, tensions and consultation times***	
Routine tests and consultation time pressure	*’Usually, we’ll examine them for* [palpable] *bladder, do a rectal examination, blood test to look at the kidneys and dip their urine for infection, diabetes as well and send an MSU off . . . ’* GP3
PSA test pressures and tensions	*’If ... they see that you’re giving them a barrier to being tested, that can sometimes, and has in one case, ended up in quite an unpleasant complaint: ”My doctor didn’t take me seriously”.’* GP4
***The nature of self-management guidance offered to men***	
Read-at-home information	*’I go to patient.info, which used to be patient.co.uk. So, I print them off that . . . It’s incredibly long . . . I think it’s eight pages printed double-sided. And that does slightly put me off printing it off. So, I think I probably have printed it one in three times, I guess.’* GP5
Lack of awareness or focus on male pelvic floor	*‘ . . . pelvic floor exercises . . . are not as well known about in men as they are with women* [and] *none of that is actually included in any of the urology clinic letters that come back from the specialist, so we’re not even seeing it in the specialists, saying, “Oh yeah, I’ve given the patient these exercises to do”, and I think, really, until that happens probably the GP’s practice won’t change.’* GP6

LUTS = lower urinary tract infection; MSU = mid-stream specimen of urine; PSA = prostate-specific antigen;

There were distinct patterns within GP descriptions of the population of men with LUTS, GPs accounts of their typical consultation and clinical care processes, and the kinds of self-management guidance that they commonly offered. These patterns indicated sites of vulnerability, where normalised clinical perceptions (of the severity of men’s LUTS for men seeking PSA tests), routine consultation pressures (leaving little time for self-management discussions during a preliminary consultation), and specific blind spots in the clinical care of male LUTS (for example, attending to the male pelvic floor), served to undermine the consistent provision of self-management guidance (or reduced this to read at home print outs).

#### Clinical conceptions of the population and needs of men with LUTS

GPs tended to discount the severity of LUTS in men who attended consultations primarily seeking PSA tests. As such, the dominant GP perception that men seeking PSA tests have few or mild (if any) symptoms, overlooked the experiences of men with severe and enduring LUTS alongside concerns about prostate cancer, as well as the likelihood that men downplay LUTS.

GPs described a principal clinical responsibility to provide a ‘biomedical’ resolution to LUTS and to address underlying aetiology through medication — in particular when symptoms were moderate to severe and causing distress. There was a sense that focusing on self-management, rather than pharmacological intervention, would be inadequate.

#### Clinical pressures and consultation time

GPs described initial male LUTS consultations that involved an essential fleet of symptom-related tests, needed to rule out alternate diagnoses. They acknowledged that delivering results over the telephone and discharging patients if PSA test results were not concerning was likely, although unintended. The lack of a routine follow-up appointment after PSA tests was linked to clinical, time-management, and appointment-volume pressures. In general, GPs described a pragmatic ‘open-door’ approach to follow-up, which assumed men would return if their symptoms were concerning or unresolved.

GPs described a degree of tension related to men’s heightened anxiety about the possibility of their having prostate cancer and the pressure to perform PSA tests. Some GPs felt that attempts to discuss self-management during a LUTS consultation with a patient focused on PSA test-seeking, would be perceived as not listening to the patient’s concerns. Patient participants agreed that discussion of self-management when they were concerned about prostate cancer and seeking a PSA test might have been unwelcome and perceived as insensitive. They also recognised that, once PSA test results had offered reassurance, they tended not to seek further GP advice, even when disruptive symptoms remained unresolved.

#### Nature of self-management information given by GPs

Several GPs referred to leaflets they printed out in consultations and/or websites containing self-management information that were sent as hyperlinks by text message to patients for them to read at home. Patients occasionally recalled receiving this kind of information; however, only rarely did they describe comprehensive or symptom-tailored advice or discussion, as opposed to more-general advice on caffeine or fluids.

GPs linked their offering of medication without extensive discussion of self-management both to their clinical responsibility to treat the underlying causes of symptoms, and to consultation time pressures and the fundamental need to exclude potentially serious alternative conditions and establish aetiology. Very few GPs described routinely discussing guidance that included pelvic floor exercises, bladder training, or urethral compression and release techniques (although some provided leaflets with this information). One GP who described regularly talking to men with LUTS about pelvic floor exercises had a specialist interest in urology, and another identified themselves as being strongly committed to promoting lifestyle approaches before prescribing medication. By contrast, some GPs acknowledged a limited focus on, and indeed knowledge of, the full range of self-management options, particularly those that focused on the male pelvic floor.

#### Integration of clinicians’ perspectives: summary

Integrating GP and patient perspectives on male LUTS highlighted the following barriers to discussing symptoms and self-management:^1^

- preconceptions about PSA-test-seeking men and symptom severity;^2^
- a biomedical focus on medication;^3^
- essential routine diagnostics that leave little time for self-management discussions, which are often replaced by leaflets or weblinks without follow-up;^4^
- the (likely accurate) perception that men may resist engagement with self-management prior to PSA testing, but follow-up is rarely initiated by either party;^5^
- where offered, self-management advice tends to prioritise diet/fluid information over pelvic floor exercises.

### Self-management intervention journey

Patient participants’ responses to receiving the TRIUMPH intervention — which have been summarised in Figure 1 and Table 1 under ‘post intervention’ — were mostly positive. Enthusiasm was strong among those who had been anxiously struggling with unresolved and poorly managed LUTS; in contrast, some men with very mild and less-disruptive symptoms were less motivated and relatively disengaged/disinterested, as were a few men with severe symptoms (for instance, a man who was considering surgery) who felt that their symptoms were beyond the reach of self-management approaches.

#### Self-management guidance

The comprehensive, targeted, self-management guidance was strongly valued by men with LUTS. They commented favourably on the booklet’s engaging, durable, user-friendly format, its readable print, plain English, and the relevant self-management approaches. They also reported having a new awareness and understanding of approaches (for instance, of the male pelvic floor and relevant exercises, of salt and caffeine reduction, of urethral compression and release) to address post-voiding dribble. Men described symptom improvements — a few were transformational, but many more were moderate — and improved quality of life. Patients associated their positivity with relief at being better informed, which reduced anxiety, increased agency in relation to symptom alleviation, and led to renewed optimism, associated with both LUTS improvements and with overcoming a stigmatising notion of loss of control and inevitable decline in ageing. A few men found the guidance unremarkable (the information was not new), unnecessary and/or unwelcome (the level of LUTS was not a great concern, the guidance recommended lifestyle changes that interfered with valued habits/choices), or hard to achieve (for example, mastering urethral compression and release, undertaking daily pelvic floor exercises). Men who experienced benefits said that the guidance should be made more widely available, and some spontaneously shared the booklet and advice they had received.

#### Intervention delivery and self-management

Some men found it helpful to talk with a trained HCP about their LUTS symptoms, and felt that follow-up calls provided additional motivation. They also appreciated having specific sections of the booklet highlighted to target their own symptoms. Others reported reading the booklet in full and trying several techniques without much of a need for oversight or motivational input; they found the material engaging and accessible, and their increased understanding and symptom control was inherently rewarding. Overall, men described positively taking the lead on their own self-management, testing the impact of different interventions on their LUTS, and evaluating the relative ease of implementation and usefulness of the different approaches in their everyday lives.

#### Integrating patients’ and HCPs’ perspectives

HCPs were interviewed, with a focus on intervention delivery. All described the intervention as accessible and straightforward to deliver. They reported:

- highly rewarding and transformational outcomes for some men;
- positive, but less-marked, improvements for many; and
- a relatively disengaged minority.

They highlighted patient participants’ prior unfamiliarity with the self-help guidance on offer:

*‘. . . through doing this study I have realised that men have been coping for years with the problems . . . to the point where they know where every single toilet* [is] *. . . Younger men, too, you know, working men who are still having to deal with it . . . They don’t realise, they think that’s it, they don’t realise that there’s help out there.’* Nurse practitioner, HCP1

*‘I don’t think the men that I spoke to realised that they had pelvic floor muscles [laughs] . . . They’re quite chuffed that they’ve been doing their exercises every day and it’s working. There was a good response.’* HCP2

‘I think they’ve seen GPs and had different referrals to things but nobody’s actually said to them about doing exercises and the gents that I saw that actually followed the booklet, improved. The majority improved and they thought it was brilliant.’ HCP3

Some expressed their own prior unfamiliarity with both the experiences of this clinical population and the self-management guidance. They noted that a focus on the male pelvic floor was novel for most men.

In summary, HCPs were positive about the intervention and their involvement in the trial led them to conclude that men with LUTS were often very poorly informed and that the TRIUMPH booklet guidance should be offered routinely in primary care. They reported unproblematic delivery in a relatively brief timeframe (a visit of around 15 minutes, with one or two brief follow-up calls).

## Discussion

### Summary

**Figure 3.**
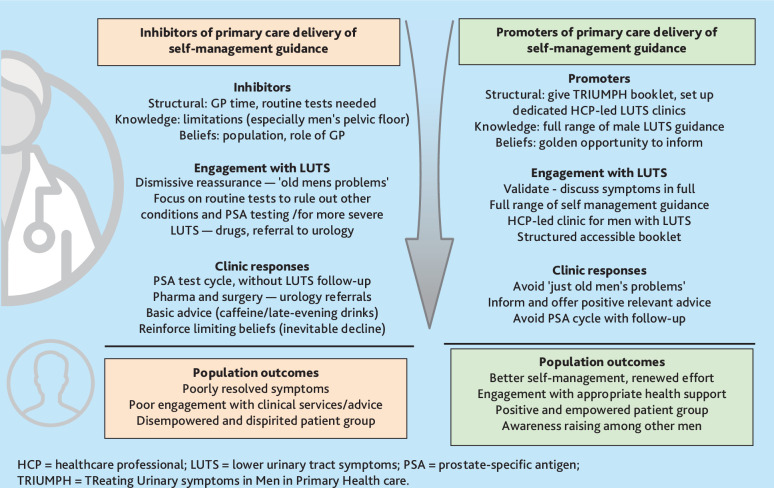
Inhibitors and promotors of the delivery of male LUTS self-management guidance in primary care.

The TRIUMPH trial found significant symptom and sustained quality-of-life improvements, without notably increased costs,^39^ critical events, or concerning side-effects.^34^ The qualitative study of the TRIUMPH intervention, with HCP-supported provision of a user-friendly booklet, supported the quantitative trial results,^32,34^ finding men to be mostly receptive, positive, and motivated to use and adhere to the guidance, with positive outcomes. Some men were highly enthusiastic and a few were relatively despondent. A few spontaneously shared their learning and booklets with peers. Beyond symptom improvements, the opportunity to be proactive and challenge perceptions of inevitable decline in ageing was strongly welcomed.

### Strengths and limitations of the study

The study’s strengths include diverse participant symptom severity, alongside clinicians’ perspectives revealing how clinical pressures may deprioritise LUTS self-management. Limitations include limited ethnic diversity and higher socio-economic status among participants, and it should be noted that there was potentially increased engagement with the intervention due to trial participation. Researcher bias was reflexively considered as a relevant concern throughout the analytic process, but positive participant experiences among men receiving the intervention contrasted strongly with continued languishing among those who did not, reinforcing the value of the intervention. As the trial predated COVID-19, post-pandemic changes to primary care could not be assessed.

### Comparison with existing literature

This work underlines the importance of focusing on LUTS that has been highlighted elsewhere,^4,17,40,41^ especially in the context of men being poorly informed, both about LUTS and about the full range of recommended self-management techniques. We found that men were often uncomfortable and unwilling to bother their GP about symptoms they deemed unworthy, because they were not sufficiently serious or life threatening; explaining the way in which both in our study, as in prior research, concerns about prostate cancer can drive primary care consultations and take precedence in LUTS consultation. This may explain the relatively high prevalence of LUTS among community-dwelling men, compared to relatively low rates of primary care LUTS consultations.^42,43^

This study’s qualitative findings challenge the notion of male LUTS as ‘just old men’s problems’ among both clinicians and men. They show that understanding and managing symptoms can reduce anxiety about prostate cancer, tying into broader PSA test concerns.^44,45^ A recent study linked rising urology referrals to both diagnostic challenges in male LUTS and to the dismissal of symptoms as age related, and called for more non-pharmacological approaches to be offered in primary care;^18^ the TRIUMPH qualitative study reinforces this need and shows how the TRIUMPH intervention can address these needs.

### Implications for research and practice

This qualitative interview study, embedded within the TRIUMPH RCT, offers insights for the primary care management of men with LUTS and opportunities to promote self-management ( Figure 3). Recommendations to promote the delivery of LUTS self-management guidance in primary care are depicted in Figure 3, identifying key factors that either inhibit or promote engagement. Supplementary Table 3 also highlights key learning for primary care that is linked to the thematic analysis. Recognising that primary care has challenging pressures, it is worth noting that, in general, men who used the TRIUMPH booklet found it easy to understand. Men also advocated strongly for the booklet to be more widely disseminated as a standalone intervention, including in social and workplace settings. The TRIUMPH trial intervention provides an opportunity to inform and empower men to more effectively self-manage their LUTS. Maximising this opportunity in primary care is warranted and the TRIUMPH booklet and intervention delivery model are available to support that process. Future research should ensure self-management support is appropriate for more ethnically diverse, disadvantaged — and often underserved — men.
